# The Signaller's Dilemma: A Cost–Benefit Analysis of Public and Private Communication

**DOI:** 10.1371/journal.pone.0013325

**Published:** 2010-10-13

**Authors:** Heiner Römer, Alexander Lang, Manfred Hartbauer

**Affiliations:** Zoology, Karl-Franzens-University, Graz, Austria; University of Minnesota, United States of America

## Abstract

**Background:**

Understanding the diversity of animal signals requires knowledge of factors which may influence the different stages of communication, from the production of a signal by the sender up to the detection, identification and final decision-making in the receiver. Yet, many studies on signalling systems focus exclusively on the sender, and often ignore the receiver side and the ecological conditions under which signals evolve.

**Methodology/Principal Findings:**

We study a neotropical katydid which uses airborne sound for long distance communication, but also an alternative form of private signalling through substrate vibration. We quantified the strength of predation by bats which eavesdrop on the airborne sound signal, by analysing insect remains at roosts of a bat family. Males do not arbitrarily use one or the other channel for communication, but spend more time with private signalling under full moon conditions, when the nocturnal rainforest favours predation by visually hunting predators. Measurements of metabolic CO_2_-production rate indicate that the energy necessary for signalling increases 3-fold in full moon nights when private signalling is favoured. The background noise level for the airborne sound channel can amount to 70 dB SPL, whereas it is low in the vibration channel in the low frequency range of the vibration signal. The active space of the airborne sound signal varies between 22 and 35 meters, contrasting with about 4 meters with the vibration signal transmitted on the insect's favourite roost plant. Signal perception was studied using neurophysiological methods under outdoor conditions, which is more reliable for the private mode of communication.

**Conclusions/Significance:**

Our results demonstrate the complex effects of ecological conditions, such as predation, nocturnal ambient light levels, and masking noise levels on the performance of receivers in detecting mating signals, and that the net advantage or disadvantage of a mode of communication strongly depends on these conditions.

## Introduction

Airborne sound as a communication channel is used in a variety of taxa, and has been particularly well studied in birds, frogs, and insects (reviews in [1], [2]). In most species, sound signals are used for communication over long distances, and the dawn and dusk choruses of many species of birds, frogs and insects are impressive displays. The sound signals have evolved primarily in the context of reproduction; in many cases it is the male who advertises its presence to a female or to potential rivals. However, due to the conspicuousness of the songs, they do not remain private to the intended receivers, but are subject to eavesdropping by unintended receivers, with potentially dramatic consequences for the signallers' survival if the eavesdropper is a parasitoid or a predator [3]–[7]. The result of this strong selection pressure often is facultative predator avoidance behaviour, such as reduced activity and feeding, or reduced or modified communication. Populations subjected to different predation regimes can rapidly diverge in their predator avoidance behaviour [8].

Since males with more conspicuous signals attract more females, but also have a higher cost of predation risk [6], [9]–[11], there exists a trade-off between sexual selection via female choice and natural selection via predators and/or parasitoids, which is most obvious in the fact that predators and parasitoids often prefer the same signal characters as females do [4], [10]–[13]. This strong selection pressure resulted in evolutionary adaptations that reduce conspicuousness to the predators [10]. One classical example is the evolution of specific anti-predator defences in a family of Neotropical katydids (Pseudophyllinae) in response to predation by foliage-gleaning bats (*Micronycteris hirsuta, Lophostoma silvicolum*) which are attracted by calling songs or other sounds involved in phonotactic activities of their prey [14]–[16]. The katydids exhibit a range of behaviours and signal characters which reduce predation by these bats, including signalling by substrate-borne vibrations. Similarly, the frog-eating bat *Trachops cirrhosus* has evolved a number of specialisations which enhance its ability to detect the low-frequency calls of one of its prey, the tungara frog *Physalaemus pustulosus*, the frogs in turn changing their signalling behaviour when they experience the predator [3], [17].

However, as emphasized by Endler [11] the conspicuousness of a signal is not a fixed property, rather it varies with environmental conditions. Signals may be attractive or not depending on the microhabitat and time of day or night, and we would therefore expect selection to act on the individual to adjust its signalling behaviour in response to these varying conditions. This has been well documented for visual signals (review in [11], [18], but reported cases in the acoustic world are rare [17], [19], [20]. In addition, each adaptive response in signalling of a prey species may produce a cascade of consequences for the cooperative communication system, including changes in the costs of signal production, in the active range of a signal, the accuracy of signal detection or discrimination by receivers etc. The sensory drive model [11], [21] considers the fact that different ecological conditions produce different trade-offs for each step in a communication system, and in order to fully understand the evolution of such a system, knowledge of the influence of the ecology for each of these steps is needed. By combining methods from ecology, behaviour, physiology, neurophysiology, and biophysics we are able to study such trade-offs for two alternative modes of communication in an insect species. We describe the changes in the mode of communication in a katydid with the lunar cycle, and the consequences for signal detection. Our results demonstrate that the net advantage or disadvantage of one or the other mode of communication depends strongly on ecological variables such as nocturnal light conditions, and thus visibility to predators.

## Results

### 1. Quantification of predation by gleaning bats

The bat species *Lophostoma silvicolum* uses the same roost as day- and nighttime shelter and returns to it between foraging bouts [22]–[24], enabling us to quantify the kind and amount of insect prey by collecting and analyzing their remains (wings, legs, ovipositors). We analyzed the roost site of one family of three individuals over a period of 86 days/year. Based on these remains, prey items could be determined in some cases to the species level. Among others 410 wings of Coleoptera and 924 wings of katydids were determined; of these 202 (22%) were *D. gigliotosi*. Thus, despite evolutionary adaptations in song redundancy and structure [14]; (see below) *D. gigliotosi* still constitutes one of the main prey of this passively listening bat.

### 2. Facultative choice of public and private mode of communication

**Figure 1:**
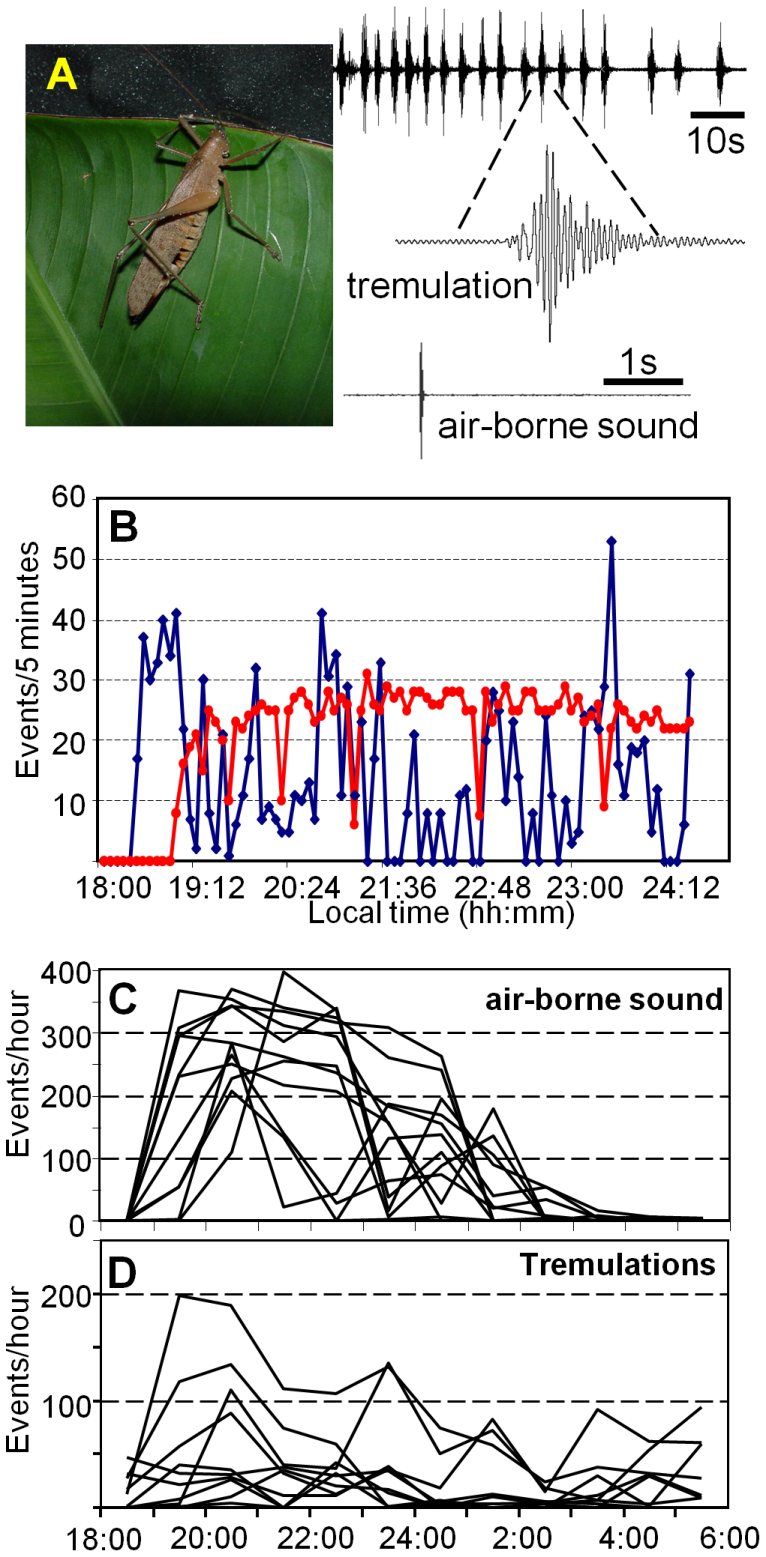
Signalling with air-borne sound and tremulations. (A) A female *Docidocercus gigliotosi* producing a series of tremulations on a plant by strong up and down movements of its abdomen; a single airborne sound pulse of a male is shown for comparison. (B) Rate of production of tremulations (blue) and air-borne sound signals (red) of a single male over the course of about 6 hours after sunset (halve moon conditions). The number of either of these two signals for a total of 11 males is shown in (C) (airborne sound) and (D) (tremulations).

In the context of mate attraction, male *D. gigliotosi* produce a calling song with elytral stridulation, consisting of a single or double syllable of short duration (24 ms for the single syllable), with a carrier frequency between 20 to 25 kHz and average sound pressure level of 80 dB at 0.5 m. The call is repeated at a low rate of 5–11/min, and therefore the duty cycle (time spent calling relative to rest) is extremely low (average of all nights 0.075%). Males and females also produce tremulation signals by shaking their body vigorously up and down in an oscillatory way without actual contact to the substrate [16], [25], [26]. The duration of a tremulation signal varies between 830 and 1300 ms (average 1110 ms ±140 ms SD); the rate varies over the period of one night (figure 1C), and between males and environmental conditions (see below). The induced substrate vibrations exhibit maximum energy at frequencies between 10 to 20 Hz, thus unusually low even for insect vibratory communication [27], [28].

Both airborne sound and tremulation signals are produced by males over the course of a night, as shown in Fig. 1B for one male during half moon light conditions. About 30 minutes after sunset, the male starts signaling with an interval of tremulation for more than 30 minutes, at a rate of approximately 30–40 events/5 min. This period of the night is the one with the highest levels of background noise in the airborne sound channel [29]. After about one hour after sunset, the male started air-borne sound production at a rate of 20–30 calls/5 minutes until midnight. At the same time, males also produced tremulations at a more irregular rate, which exceeded the call rate occasionally up to 50 events/5 minutes. There was a high variation between males with respect to the total amount of signaling (Fig. 1C, D). For example, more than half of the males showed overlapping intervals of tremulation and sound production, where a sound signal was followed by tremulation after only 200 ms, whereas others switched between exclusive intervals of calls or tremulations, with no temporal overlap. Moreover, males also varied with respect to the relationship between double- or single-syllable chirps; some males always called with double syllables, others only with single syllables, or with both.

#### Influence of the lunar cycle on signaling

**Figure 2:**
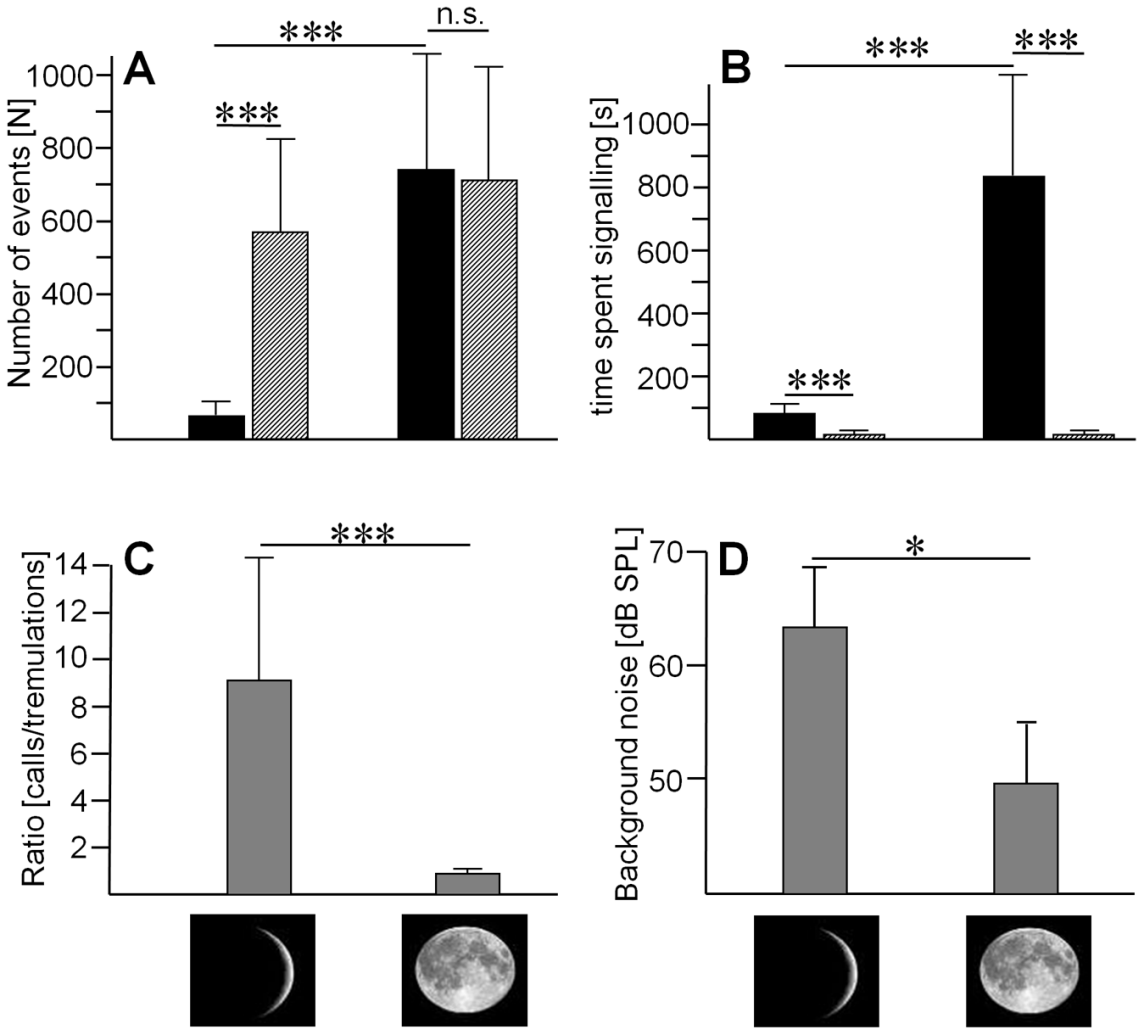
The amount of signalling in the private and public mode depends on nocturnal light conditions. The amount of calling vs. tremulations (hatched and black bars, respectively) (A), the time spent signalling (B) and the ratio of calls vs. tremulations (C) differs significantly between new-moon and full-moon nights. (D) The overall sound-pressure level is significantly reduced by 13.6 dB under full-moon conditions compared to under new-moon conditions.

The relationship between air-borne sound signals and tremulation signals correlated significantly with the lunar cycle, and thus the ambient light conditions at night (Fig. 2A). Under new-moon conditions (between 0–25% of the moon's visible disk illuminated), males signaled on average almost 600 times by airborne sound compared to less than 100 times by tremulation, but under full-moon conditions (more than 75% of the moon's visible disk illuminated) signaling by tremulation is increased significantly to more than 700 times, whereas calling by sound remained unchanged (Mann-Whitney-rank-sum test; p<0.0001).

Thus the ratio of calling/tremulation shifted from 9.2 to 1.1 under full moon conditions (p<0.0001; Fig. 2C). The total average time signaling with tremulation increases from 80 s under new-moon conditions to 823 s under full-moon, whereas signaling time using air-borne sound is almost unchanged (14.2 s compared to 17.1 s; Fig. 2B). Under higher illumination at night males spent significantly more time signaling in the more private compared to the public mode of communication. The duty cycle (“on-time” of signaling relative to rest) of both signal types is very low, but differs under the two ambient light conditions: for tremulation signals, duty cycle increases from 0.27% to 2.01%, but remains almost constant at 0.07% and 0.08% for calls, respectively.

These observations are consistent with *D. gigliotosi* having a conditional strategy of signaling, where fairly cryptic (i.e. short duration, low redundancy) airborne sound production is replaced by the even more private mode of communication with tremulations under light conditions which increase vulnerability due to successful predation by visually hunting predators [30], [31]. This interpretation is supported by a comparison of background acoustic noise levels on new-moon and full-moon nights, which are reduced by 13 dB in the latter (Fig. 2D). Since most of the acoustic background noise is due to signaling of insects, the reduction in full moon nights must be due to a significant partial or complete reduction in sound production of a number of species and/or individuals.

#### Energetic costs associated with both types of signalling

Whereas the advantage of private signalling in the face of potential eavesdroppers to airborne signals appears obvious, signalling with tremulations might be more costly in energy terms. The production of CO_2_ was therefore recorded in a small metabolic chamber while the insect was either calling or tremulating. This allowed quantifying the respective energetic costs associated with either form of signalling. The average amount of CO_2_ production associated with one tremulation and one acoustic signal was determined; signalling by tremulation produces on average 4.89 µl CO_2_/signal compared to 0.73 µl CO_2_/sound signal (p<0.001; Mann-Whitney rank sum test, n = 18). Due to the different amount of tremulation versus calling (see above), an average full-moon night with increased tremulation rate is therefore energetically more demanding than a new-moon night (2807 µl CO_2_/night compared to 893 µl CO_2_/night).

#### Signal transmission and active range of private and public signals

**Figure 3:**
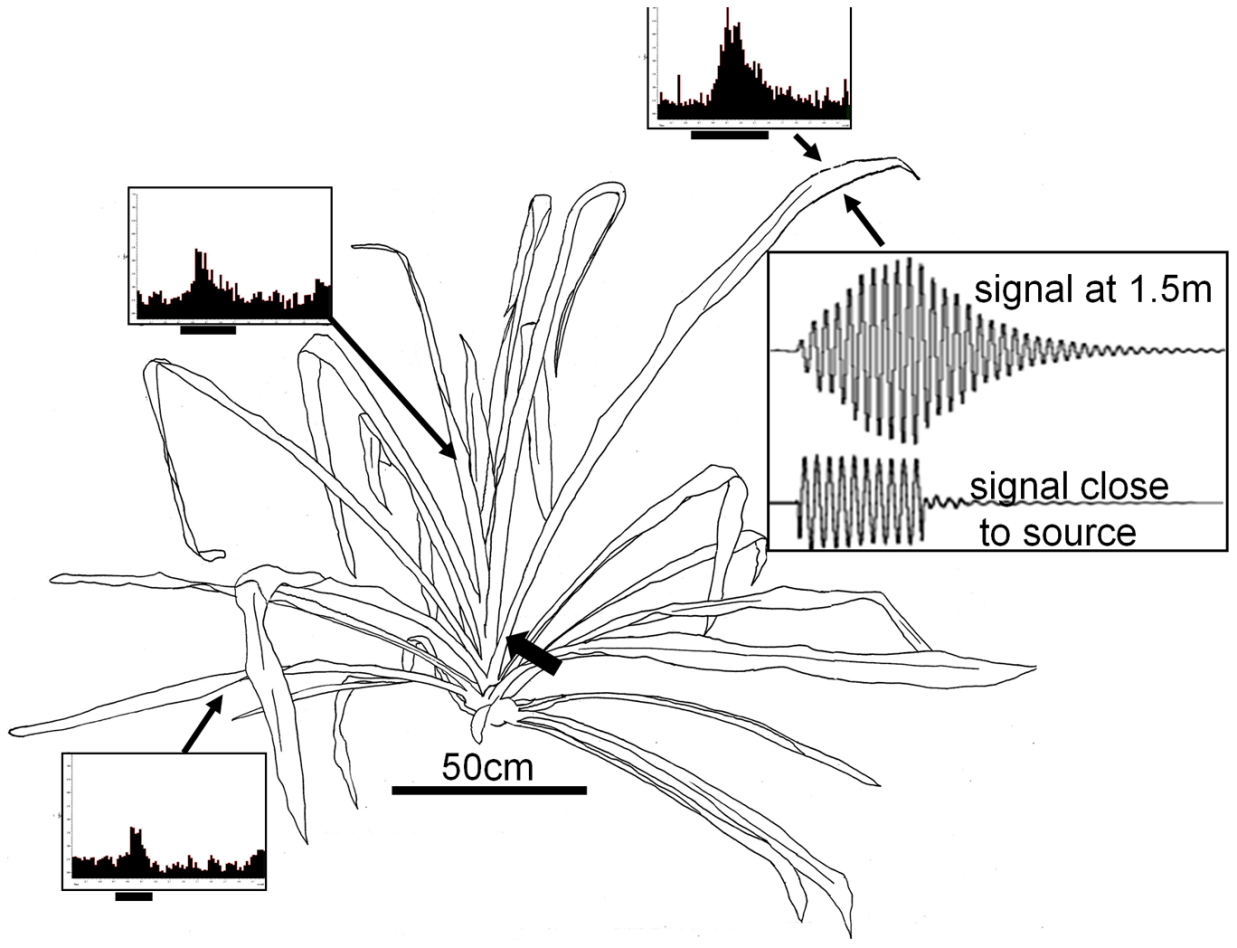
Transmission and perception of substrate-borne vibrations along *Aechmea magdalenae*, the roost plant of the katydid *D. gigliotosi*. When one leaf of the plant is stimulated with a sinusoidal stimulus at 10 Hz (at the position indicated by the large arrowhead), the induced vibrations of the plant differ substantially close to the source (distance 5 cm) and at a distance of 150 cm. Note the slow increase of acceleration amplitude after stimulus onset, and corresponding decrease at the end, indicative of resonant properties of the plant at this frequency. Three PST-histograms of responses of vibration receptors are shown for three positions on the plant (arrows), when the stimulus was a male tremulation induced at the position of the arrowhead. The receptor response was largest for the position at the end of the same leaf, where the acceleration amplitude was high, but suprathreshold responses were also observed on other leaves. For further information see text.

We measured the active range of airborne sound signals using a “biological microphone” technique [32], [33]. A conspecific sound signal was broadcast in the understory of the rainforest (80 dB SPL at 0.5 m distance from speaker), and the maximum distance at which the nervous system of a receiver responded to the signal was recorded. In a total of 10 such experiments, this range varied between 22 and 35 m (mean 27.4 m ±4.3 m SD). The active range of the tremulation signal was determined in a two-step process. First, the transmission properties of the preferred roost plant of *D. gigliotosi*, the bromelid *Aechmea magdalena* were examined for various frequencies by stimulating the plant at the base of the calyx with a vibration exciter, and recording the transmitted substrate vibrations along the leaves using laser-vibrometry (figure 3A). Apparently, the leaves show resonator properties for frequencies between 10–15 Hz, where the signal amplitudes are not only least attenuated, but often enhanced after transmission with an increase and decay in amplitude typical for resonators (compare signal close to the source and at 1.5 m; figure 3). This range of enhanced frequencies corresponds well with the maximum energy in the tremulation signal of the katydid at 13 Hz.

Next, we used a pre-recorded tremulation signal as playback to stimulate the plant (*Aechmea magdalenae*) with a vibration exciter, and recorded the signal at various positions along single leaves, and on different leaves, after transmission. The transmitted signals were then used in a consecutive neurophysiological approach as playbacks to stimulate the sensory system of the katydid and determine whether these signals would activate sensory receptors above threshold. Examples of such responses from multiunit recordings of the leg nerve, containing fibres from the complex tibial organ (including the vibration-sensitive subgenual organ), are shown in Fig. 3 as peri-stimulus-time-histograms. Irrespective of the position on the plant where the transmitted tremulation signal had been recorded, each signal exhibited amplitudes which would have induced suprathreshold responses in the vibratory system of the insect, if it were standing at these positions.

#### Reliability of detection by the receiver

**Figure 4:**
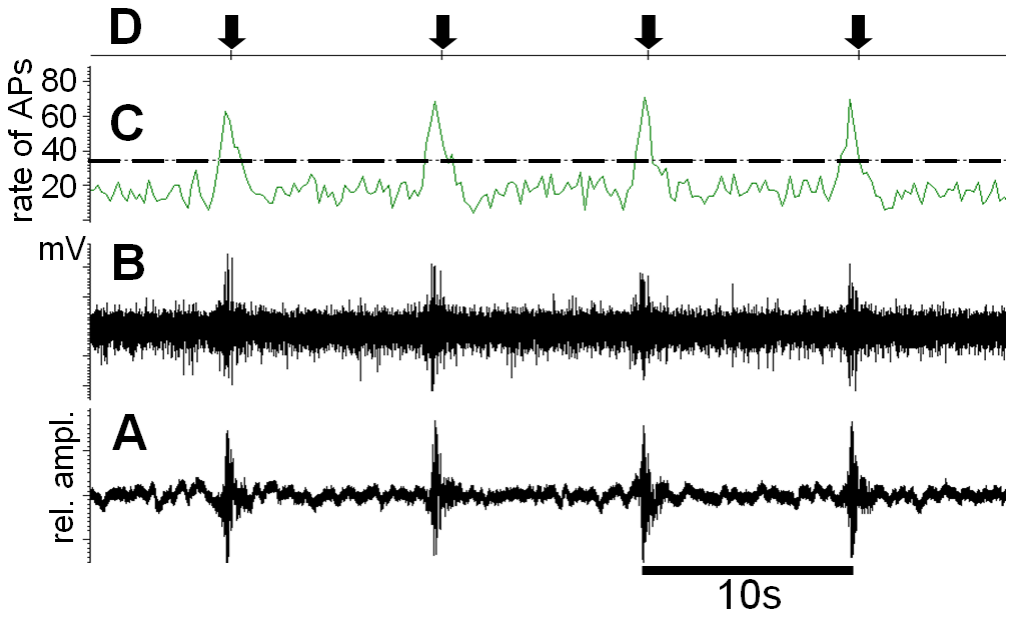
Reliability of detection of the tremulation signal. (A) Tremulation signal in the background noise of a leaf of *A. magdalenae*. (B) Action potential activity of the frontal leg nerve carrying fibres of the subgenual and hearing organ. (C) Instantaneous spike rate analysis of the recording shown in (B). Stippled line indicates threshold for detection (2 times SD of spontaneous activity). (D) Result of signal detection (arrows indicate “hits”).

One aspect common to all kinds of communication in different modalities are the constraints imposed by background noise, resulting in reduced signal-to-noise-ratios, which limit the active space of a given signal [34], [35]. Whether or not a signal is effective in eliciting a response in the receiver under masking noise conditions can be determined either directly via its behaviour, or indirectly, by analysing the sensory system under natural conditions. Afferent activity of receptors either sensitive to air-borne sound or to substrate vibration was therefore recorded under natural noise conditions in the respective transmission channel, and the reliability of detecting the signal determined. Fig. 4 gives one example mimicking a situation for a receiver placed on a leaf of the plant *Aechmea magdalenae*, when a male is tremulating within the calyx of the plant (where males have been observed tremulating in the first hours of nocturnal activity). With each tremulation signal there is a strong increase in spike rate in the summed receptor activity, and assuming a threshold of detection which is two times above the standard deviation of the average spike rate during the time without stimulation, one can calculate the rate of signal detection for this kind of signal. The summary for signal detection (8 preparations; total time of analysis 303 min; 1866 signal presentations) was 94.8±5.2% hits. At the same time, “false alarms” would occur when the spike rate reached this criterion but there was no signal present. A mean of 1.1±0.33 false alarms/minute was found. Thus the detection for the tremulation signal was very reliable.

Similar experiments were performed using recordings of summed action potential activity of air-borne sound receptors, with the preparations placed in the nocturnal rainforest and receiving conspecific calls at a sound pressure level either 10 dB or 20 dB above the hearing threshold. The summary for detection of the air-borne sound signal (10 preparations; total time of analysis 223 min; 3738 signals presented) was 83.9±17.3% hits (10 dB above threshold), 89.3±13.8% hits (20 dB above threshold), and a mean of 16.5±7.5 false alarms/minute (10 dB above threshold), 15.0±6.3 false alarms/minute (20 dB above threshold; figure 4). The amount of hits was not significantly different for the two sound levels presented (p>0.05, Mann Whitney rank sum test). Thus, the same analysis of signal detection as done for the tremulation signal revealed a reliable rate of signal detection for the airborne sound signal, however, the rate of false alarms was rather high. Although in both series of experiments signal detection was analysed under natural noise conditions, the private vibratory channel is much less noisy for the sensory system of the insect, and provides a more reliable detection, compared to the air-borne sound channel.

## Discussion

Predation is one of the strongest selection pressures, and its importance in the evolution of adaptations, such as cryptic coloration, chemical and other defences etc. has long been recognised [5], [6], [36]. Katydids represent the primary protein source for many vertebrates and invertebrates. The nocturnal lifestyle of most katydid species and the selection of certain roost sites is a response to visually searching predators during the day [37]–[39]. At night, their dyadic communication system is exploited by certain bats, which act as unintended receivers and eavesdrop on the katydids' mating calls. The strong reduction in the call duty cycle and the cryptic life style of *D. gigliotosi* and other katydids are considered evolutionary adaptations to this predation pressure [6], [14]. The alternative use of tremulation signals as a private communication channel should be particularly effective because this predator is unable to detect such signals (for a similar case of a private channel using UV light in visual communication see [40]).

Our results on the insect remains at a roost site of a family of *L. silvicolum* indicate, that despite these adaptations *D. gigliotosi* constitutes more than 20% of all katydid prey (see also [41]). For the individual insect, however, the predation pressure may not be constant over its lifetime, and if it is sensitive to changes in predation risk it should adapt its decisions for the amount of public and private signalling to these changes. The nocturnal light level is an ecological determinant of risk, since it influences the visual ability of predators [5], [42]. Indeed, *D. gigliotosi* varies the relative amount of calling and tremulation with the moon cycle; in periods of high visibility in the rainforest understory there is a shift to more private signalling (Fig. 2). Remarkably, the shift to private signalling under high nocturnal light levels is unrelated to the bat predator eavesdropping on the airborne sound signal, since these bats have been shown to reduce their foraging activity at full moon as well [24]. Such reduced foraging by bats has been discussed as a secondary response to the reduced availability of prey species, rather than predation on the bats by their own predators.

We have also shown that during full moon conditions the background noise level in the nocturnal rainforest is reduced by 13 dB on average. Since species such as *D. gigliotosi* with a strongly reduced song duty cycle contribute very little to the background noise, the significant noise reduction under full moon light conditions must be due to a partial or complete reduction of sound production of many species of insects and frogs. Paradoxically, male *D. gigliotosi* would have a double advantage when using airborne sound under these conditions: first, they would not incur the risk of predation by eavesdropping bats, which are much less active during these nights [24], and second, the reduced masking noise would allow a better detection of their signals by receivers (see below). We assume that the main reason why these males nevertheless reduce the amount of public signalling is, that predation risk does not only include the costs due to increased conspicuousness when displaying/signalling, but also the risks involved in mate searching activities [36]. Females performing phonotaxis over considerable distances to calling males would pay the costs of predation, because movement is the best stimulus eliciting attention in the visual and auditory system of nocturnal predators [43], [44]. Thus, if females are less likely to perform phonotaxis by either walking or flying during full moon, males, as a consequence, should invest less in public signalling. By contrast, communication by tremulation happens over relatively short distances (see below) and partly on preferred plants serving as roost sites [39] where predation risk even during full moon is reduced, and thus the switch to more private signalling appears adaptive for males and females.

We do not argue that switching to the private mode of communication does completely remove predation risk. Predators or parasitoids equipped with vibration sensitive receptors, and attached to the same substrate as the signaller may detect and home in on the tremulation signal, as does an egg parasitoid eavesdropping on sexual vibratory signals of stink bugs [45]. Spiders, with their high sensitivity for substrate vibrations [46], [47] are also potential candidates for eavesdropping on tremulation signals of *D. gigliotosi*, as already suggested by [16]. In our survey on the site fidelity of *D. gigliotosi* for the bromelid *A. magdalena* we regularly found some plants occupied by spiders of the genus *Cupiennius*, which prey upon katydids [39]. However, the density of spiders was relatively low with about 1/25 plants. Thus, despite notes of the vast abundance of predatory spiders for insects [48], it appears that when *D. gigliotosi* switches from air-borne sound to tremulations under some ecological conditions it escapes a stronger predation pressure in the public mode of communication, than it suffers from predation in the private mode.

Energy is the basis of trade-offs for the evolution of many traits (for an example in crickets see [49]). Because acoustic signaling in small animals like insects is energetically demanding (mainly due to the low efficiency with which metabolic energy is converted to acoustic power [50], [51], the difference in the energetic costs of calling and tremulation could play a role in a decision for the facultative use of one or the other of these signals. Our measurements of the energetic costs for an air-borne sound signal compared to tremulation, and the calculation for the average rate of both signals for a full-moon compared to new-moon night demonstrate a 3-fold increase in energetic costs when the insect increases the rate of communication in the private channel. However, in comparison with the energetic demands associated with locomotion during walking or in flight, both types of signals are rather inexpensive [50]. We also have to consider that energetic limits on signaling could depend on how easily energetic stores can be replenished on a daily basis [50]. If energy-rich food is sparse, energy reserves may indeed limit signaling. In a laboratory study on a synchronizing katydid the decrease in body weight after several singing bouts during the night was fully compensated after only two hours feeding on lettuce [52]. We would therefore argue that despite the increase in energetic demands from tremulations, this would not represent a major constraint for producing these signals.

The main evolved function of acoustic signal production in insects is to attract mates and to engage in male-male competition [1], [2]. Thus, the area where a signal can be detected by receptive mates is critical for the ultimate reproductive success of the signaller. This area is defined as “broadcast area” [53] or “active space” [54]. Theoretically, three parameters define the active space of a signal: the intensity of the signaller, the degree of attenuation of signal amplitude during transmission, and the hearing threshold of the receiver. A switch from air-borne sound to tremulation should be associated with changes in the active space of the signal, since the perception of tremulations is limited to the substrate to which both sender and receiver are attached, whereas airborne sound can be transmitted over considerable distances, even if the transmission channel includes scattering vegetation. The preferred plant of *D. gigliotosi* is the bromelid *Aechmea magdalena*, where many undivided leaves extruding from the calyx can be as long as 3 meters [39], [55]. It was not clear previous to our study, though, whether a vibratory signal produced by a male somewhere on the plant is strong enough (suprathreshold) to be detected by female receivers.

Our results, using neurophysiological methods clearly demonstrate that this is indeed the case: stimulating the plant with a male tremulation signal within the calyx (where males were often found) result in perceived signals which elicited clear suprathreshold responses in the leg nerve, most likely in receptors of the subgenual organ of the complex tibia organ described for Ensifera [56]. This was true for any position of the receiver on the plant (Fig. 3), so that a tremulating male will be able to signal its presence to females, once they have contact with any leaf of the plant. However, although this is one of the largest active spaces ever reported for a vibratory signal [27], [28], [57], it is still considerably smaller than that of the airborne sound signal. Detection distances between 22 und 35 m (mean 27.4 m ±4.3 m SD) appear rather high since the male song uses high frequencies around 25 kHz, and such high frequencies suffer from strong excess attenuation in scattering vegetation [33]. Yet, the understory of the tropical rainforest on BCI does not include dense vegetation and is a rather open space for sound transmission, including high sonic and ultrasonic frequencies.

**Figure 5:**
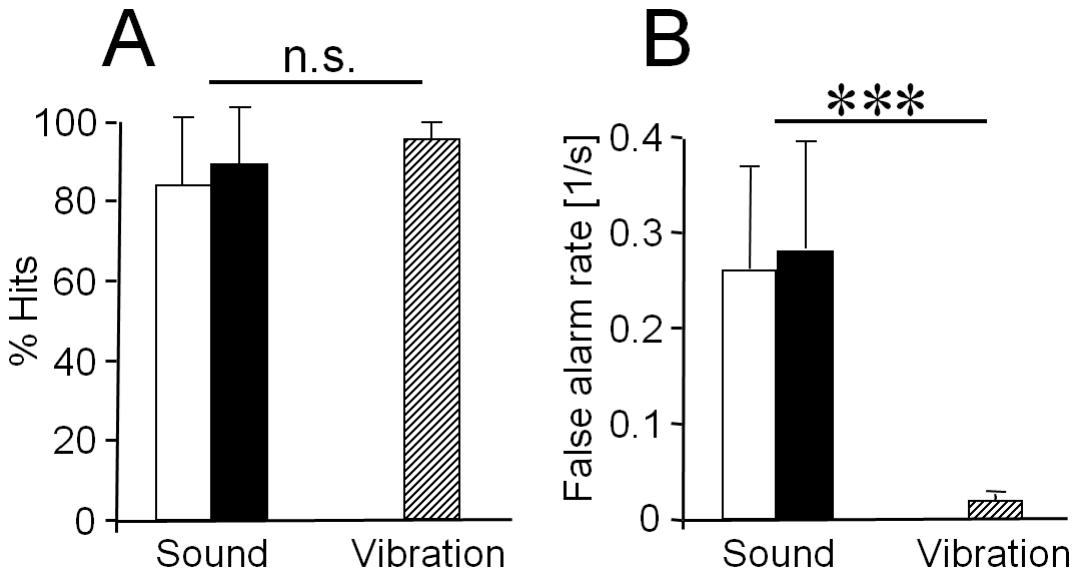
Comparison of “hits” (A) and “false alarms” (B) achieved in the two modes of communication. The air-borne sound stimulus was either 10 dB or 20 dB above threshold at the position of the receiver (white and black bars, respectively). The tremulation signal amplitude was about 20 dB above threshold. For further explanation see text.

However, since the definition of the detection distance also includes the sensitivity of the receiver and its ability to detect a given signal, we have to consider the performance of receivers for both modes of communication under the existing levels of background noise. In recent years increasing attention has been paid to the impact of natural background noise in different modalities (review in [35]), and applications of game theory [58]–[60] as well as signal detection theory [61], [62] demonstrated the importance of errors as a result of noise for the evolution of a communication system. Our results have shown, using the response of the afferent nervous system under natural background noise as an indicator, that signal detection for the 24-ms signal of *D. gigliotosi* reached values of about 85–90% hits, depending on broadcast amplitude. Values for the tremulation signal (1100 ms in duration), again determined under the nocturnal background noise vibrations of the plant, have been close to 95% hits. The major difference, however, was the amount of false alarms in the two modes of communication, which is one type of error in signal detection producing a response when the appropriate signal was absent. These false alarms occurred at a high rate of 0.25 to 0.3/s for air-borne sound signals, but more than an order of magnitude less for tremulations (Fig. 5). These results from recordings of the sensory receptors in both modes of communication were corroborated in experiments where we used outdoor recordings of the action-potential activity of a second-order interneuron and its burst responses to both playback stimuli and to nocturnal rainforest background noise [63]. An unsupervised clustering algorithm applied to the burst activity often clustered the bursts in response to the short stimuli of *D. gigliotosi* together with bursts elicited by background noise, whereas this never or rarely happened with bursts which resulted from responses to longer or more complex, temporally modulated, stimuli. If the task for the “psychology of receivers” [64] is not only the detection of the appropriate signal, but to discriminate between two or more male signals differing slightly in their properties, this is even more demanding. In humans, error levels increase for tasks that require discrimination compared to those requiring detection only, and subjects failed to discriminate when they correctly detected a signal [65]. Even under no background noise at all discrimination performance decreases as the number of choices increases, as evident in the consistency of preferences of female anurans [66]–[68].

Clearly, with respect to the task of signal detection and/or discrimination, the public mode of communication using air-borne sound suffers from high levels of background noise and the resulting errors, and is at a disadvantage compared to the private mode of communication, since high levels of further signal processing would be required to correctly reject excitation in the sensory system as a result of noise. Natural selection through predation by passively listening bats appears to have forced males in this species to produce extremely low-redundancy, airborne signals [14], whereas high duration tremulation signals in low background noise do not show these kinds of limitations for receivers. Table 1 summarizes the advantages and disadvantages of the two modes of communication in this insect. It is evident that none of them is free of disadvantages, and as predicted from the sensory drive hypothesis, the use of one and the other produces trades-offs where the net benefit strongly depends on the ecological variables. The behaviour of the studied katydid would indicate that it accounts for these variables.

**Table 1:** Summary of the costs and benefits of the public and private mode of communication in *D. gigliotosi*.

	Air-borne sound signal	Tremulation signal
**Predation**	high	low (?)
**Energetic costs**	low	high
**Active range**	22–35 m	about 4 m
**Signal detection**	difficult; many false alarms	highly reliable

## Materials and Methods

### Ethics statement

The experiments reported in this paper comply with the current animal protection law in Austria, and with current Panamanian laws. According to these laws, studies on insects do not require approval by a review board institution or ethics committee.

Most methods have been described in detail in [32], [52]; and are only briefly summarized here.

### Animals and study site

The study was conducted on Barro Colorado Island (BCI; 0°09'N, 79°51'W), Panama, in February/March and June/July 2002, 2003, and 2005, in the dry season and at the beginning of the rainy season, respectively. We studied *Docidocercus gigliotosi*, a pseudophylline katydid which is one of the most common katydids on the island [41].

### Signalling activity

The signaling activity by airborne sound and substrate vibration of isolated males was continuously recorded during the night in a rainforest gap, at different times within the lunar cycle. Males were collected on the island and kept in containers with other males. One day prior to the measurement, they were isolated in small boxes (size 10×10×15 cm) made of transparent plastic. A small elektret microphone was placed inside the box, and an accelerometer (Rion 4440) attached to one wall. About one hour before sunset the box with the male was placed in a large gap in the rainforest, so that moon light had full access to the male. The outputs of the accelerometer with connected amplifier (Vibration meter Rion UV-05), as well as the microphone, were recorded on separate channels of a Maclab/Powerlab 4e data acquisition system (AD Instruments Pty Ltd) at a sampling rate of 5 kHz. Each male was tested for one night only; a total of 26 males were used at different lunar cycles over 2 months.

### Signal transmission, active range and noise

We quantified the effect of lunar cycle on background noise level in the airborne sound channel with a continuous sound recording system (described in detail by Lang et al. 2005). The system consisted of a sound level meter (CEL 414 plus attached CEL-296 digital filter - settings: A-weighting; slow time constant) with a condenser microphone (LD 2540, Type 4133, range 4 Hz–45 kHz). The set-up was protected from humidity and rainfall and heated to 2°C above ambient temperature with an infrared bulb to prevent fogging of the microphone membrane. Sound recordings were made in nights at different phases of the lunar cycle in February, May and June, as well as from the end of October to early December 2002. Background noise in the vibratory channel was recorded on the preferred roost plant of the insect, the bromelid *Aechmea magdalenae* [39]. Recordings were made with a laser vibrometer (OFV-353 sensor head and OFV-2200 controller and PDV100; Polytec, Waldbronn, Germany) or accelerometer (Rion 4440) and a data acquisition system (Maclab/Powerlab 4e; AD Instruments Pty Ltd) at a sampling rate of 10 kHz for later playbacks (see below).

### Determination of the active range for both signal types

The active range of the airborne sound signal was determined using a method described in detail by [32], [33]. A speaker (DynAudio D21/2; frequency range 2–40 kHz) was used to broadcast the conspecific sound signal through the understory of the rainforest at a height of 1 m. A portable neurophysiological preparation with extracellular recordings of action potentials of a sound sensitive interneuron (the so-called omega-neuron) was moved away from the speaker until the neuron just responded at threshold to the signal. This procedure was repeated four times with the speaker broadcasting into different directions (N = 8). To determine the active range of the vibratory signal the neurophysiological preparation was modified to record multi-unit action potential activity of vibration receptors in the leg nerve of *D. gigliotosi*. The front leg of the insect was fixed with a tarsus in a normal (inverse) standing position to the cone of a minishaker (4810; Bruel & Kjaer). Stimulus presentation was controlled via Cool Edit Pro (2.0, Syntrillium). Stimuli have been prerecorded with the laser-vibrometer at the various positions of the plant after stimulating the plant with the tremulation signal via the minishaker (Fig. 3). The multi-unit response of receptor fibres was recorded 10 times and peri-stimulus-time-histograms (PSTH) were calculated in order to determine suprathreshold responses to the stimulus (bin width 5 ms).

### Signal detection under natural conditions

To characterize the receivers' ability to detect the conspecific airborne sound signal under natural conditions, we monitored the multi-unit action potential activity of auditory receptor fibres in the prothoracic ganglion in the first four hours after sunset in the rainforest. The portable neurophysiological preparation was placed at a distance of 10 m from a speaker broadcasting the conspecific sound signal at a rate of 0.1/s, with an SPL adjusted to either 10 dB or 20 dB above the threshold of the preparation. A total of 11 preparations were recorded and analysed for a time of one hour each. A signal produced a burst of multi-unit action potentials, which was considered to be detected (hit) when the spike rate exceeded a critical value of two times the standard deviation of the spontaneous spike rate for 20 ms (see Fig. 4). Similarly, a burst of action potentials following the same criteria was considered a false alarm, when it was not associated with a stimulus, i.e. induced by noise in the air-borne sound channel.

A similar approach was adapted for the vibratory channel, by using the multi-unit action potential activity of vibration receptors in the leg nerve. The front legs of the preparation were attached to a vibrator broadcasting vibratory noise for one hour. This noise had been pre-recorded with a laser vibrometer from a bromelid in the nocturnal rainforest on one leaf at a distance of 1 m from the calyx (total duration 10 hours in 3 different nights). A continuous section of one hour with the maximum acceleration occurring in the three nights was used for playback. The noise was digitally mixed with a recording of a conspecific tremulation signal every 10 seconds (using audio software (CoolEdit Pro, Syntryllium Inc.; see Fig. 4). Both the signal and the background vibration noise were played back through a D/A board at a sampling rate of 10 kHz. In these experiments, the sensitivity of the preparation to airborne sound was reduced by plugging both acoustic spiracles, and the tympana in the forelegs, with petroleum jelly. This rendered the threshold to sound well above 70 dB SPL, and thus above the background noise in the sound reduced chamber, where the preparation was placed. Similar signal detection criteria as for the analysis of airborne sound signals were used, except that the spike rate had to exceed the critical value for a longer duration of 200 ms, since the tremulation signal lasts much longer.
